# The intergenerational transmission of intimate partner violence in Bangladesh

**DOI:** 10.3402/gha.v7.23591

**Published:** 2014-05-23

**Authors:** Towfiqua Mahfuza Islam, Md. Ismail Tareque, Andrew D. Tiedt, Nazrul Hoque

**Affiliations:** 1 Department of Health Care Management and Planning, Graduate School of Medical and Dental Science, Tokyo Medical and Dental University, Tokyo, Japan; 2 Department of Population Science and Human Resource Development, University of Rajshahi, Rajshahi, Bangladesh; 3 United States Department of Justice, Washington DC, USA; 4 Hobby Center for Public Policy, University of Houston, Houston, TX, USA

**Keywords:** intimate partner violence, inter-parental physical violence, women, Bangladesh

## Abstract

**Background:**

A number of individual risk factors for intimate partner violence (IPV) have been identified in Bangladesh. However, the etiology of IPV, intergenerational transmission, has never been tested in Bangladesh.

**Objective:**

We examined whether witnessing inter-parental physical violence (IPPV) was associated with IPV to identify whether IPV passes across generations in Bangladesh.

**Methods:**

We used nationally representative data of currently married women from the Bangladesh Demographic and Health Survey-2007. Variations in experiencing IPV were assessed by Chi-square tests. Logistic regression models were fit to determine the association between witnessing IPPV and different types of IPV against women.

**Results:**

One-fourth of women witnessed IPPV and experienced IPV. After adjusting for the covariates, women who witnessed IPPV were 2.4 (95% confidence interval [CI]: 2.0–2.8) times more likely to experience any kind of IPV, 2.5 (95% CI: 2.0–3.0) times more likely to experience moderate physical IPV, 2.3 (95% CI: 1.8–3.0) times more likely to experience severe physical IPV, and 1.8 (95% CI: 1.4–2.3) times more likely to experience sexual IPV. Age, age at first marriage, literacy, work status, wealth, justified wife beating, and women's autonomy were also identified as significant correlates of IPV.

**Conclusions:**

This study's results indicate that IPV passes from one generation to another. We make recommendations for preventing IPPV so that subsequent generations can enjoy healthy, respectful, nonviolent relationships in married life without exposure to IPV in Bangladesh.

Violence against women is ubiquitous. The number of studies examining violence against women has increased dramatically over the past three decades, and violence against women has gradually become a public health priority (1). Intimate partner violence (IPV) is one of the most common forms of violence against women worldwide (2). In Bangladesh, several studies identified a number of individual risk factors for IPV. For example, studies have found that the risk of experiencing violence was significantly higher among younger women (3, 4), less educated women (5, 6), women with less educated husbands (5), women living in poor households (5), and women who believed their husbands were justified in beating them in certain circumstances (7). However, the etiology of IPV, intergenerational transmission, has never been tested in Bangladesh.

With roots in social learning theory (8, 9), the intergenerational transmission of violence theory has suggested that children learn violence from watching their parents and intimate violence tends to run in families (10). When children witness violence between parents, they receive direct messages about the appropriateness of marital aggression. Black, Sussman, and Unger (11) have found evidence in support of the intergenerational transmission of violence and suggested that adults, or individuals aged 18–27, may have been negatively influenced by witnessing inter-parental violence. Many researchers have also sought evidence that IPV passes from one generation to another, such that children who were exposed to violence in their families of origin, either by experiencing childhood violence or witnessing inter-parental violence, were more likely to use violence in their families as adults than children who were never exposed to familial violence (12). In the United States, having witnessed violence as children was reported to be a significant factor for being victimized in terms of psychological IPV (13). In Romania, a positive correlation between witnessing inter-parental violence and accepting such violence in one's IPV was found (14). In urban Thailand, a direct association between childhood exposure to inter-parental physical violence (IPPV) and subsequent psychological and physical victimization in adulthood was found among married women (15). In Nigeria, women who ever witnessed IPPV were reported to have tolerant attitudes toward IPV against women and were more likely to be abused by spouses (16). In light of this evidence, we stress the importance of IPPV in exposing women to the belief that violence is a normal practice in married life. Therefore, we examined whether witnessing IPPV was associated with IPV among a nationally representative sample of married Bangladeshi women in order to test whether IPV is passed down from one generation to another.

Finally, we made policy recommendations, guided by the results, that center on educational programs targeted at both genders. We underscore that the focus of these programs is men since men are the most likely violent offenders and men of all ages play a significant role in tolerating IPV thereby perpetuating intergenerational transmissions.

## Materials and methods

### Data

We used data from the Bangladesh Demographic and Health Survey (BDHS)-2007. Using face-to-face interviews, BDHS-2007 collected nationally representative data on demographic and health indicators from ever-married women, aged 15–49 in private dwelling units in Bangladesh. The survey was conducted under the authority of the National Institute for Population Research and Training (NIPORT) of the Ministry of Health and Family Welfare of Bangladesh (17). The survey instrument was implemented by Mitra and Associates, a Bangladeshi research firm located in Dhaka. Macro International Inc., a private research firm located in Calverton, MD, USA, provided technical assistance to the survey as part of its international Demographic and Health Surveys program. The US Agency for International Development (USAID)/Bangladesh provided financial assistance.

The BDHS-2007 used the sampling frame provided by the list of census enumeration areas with population and household information from the 2001 Population Census. It administered five questionnaires: a Household Questionnaire, a Women's Questionnaire, a Men's Questionnaire, a Community Questionnaire, and a Facility Questionnaire. A violence module on the Women's Questionnaire was used to collect data on IPV against women. A total of 10,819 households were selected for the sample based on a two-stage stratified sample of households; all ever-married women aged 15–49 (11,178 individuals) were identified in selected households, and 10,996 were interviewed, for a response rate of 98.4%. Four quality control teams ensured data integrity; each team included one male and one female. In addition, NIPORT monitored fieldwork with another set of quality control teams (17). Only one woman from each selected household was interviewed with the domestic violence questionnaire. If there was more than one eligible respondent in the household, the respondent was selected randomly through a specially designed simple selection procedure, utilizing the Kish Grid (18). Based on the criterion of choosing one woman from each selected household, 4,489 ever-married women were found eligible to respond to the domestic violence module. Only 22 had to be excluded owing to lack of privacy (husband, other male or female were present in the household during the interview period). Thus, the ever-married sample was reduced to 4,467. Out of this number, currently married women were chosen and the sample size was further reduced to 4,193. Finally, the cases with missing observations for all the variables used in this study were excluded, bringing the sample size to 3,910.

### Outcomes

The survey measured spousal violence with a shortened and modified Conflict Tactics Scale (CTS) (19, 20). Among currently married women, the questions were asked with reference to their husbands, with the goal of estimating IPV prevalence for the 12 months preceding the survey. Women were asked the following eight questions:

Does/did your (last) husband ever do any of the following things to you:

- Push you, shake you, or throw something at you?
- Slap you?
- Twist your arm or pull your hair?
- Punch you with his fist or with something that could hurt you?
- Kick you, drag you, or beat you up?
- Try to choke you or burn you on purpose?
- Threaten or attack you with a knife, gun, or any other weapon?
- Physically force you to have sexual intercourse with him even when you did not want to?

The health impacts of the above violent acts are not limited to physical injury; long-term effects include depression, mental disorders, suicide attempts, chronic pain syndromes, unwanted pregnancy, HIV/AIDS, and other sexually transmitted diseases (21, 22). The above eight forms of violence could also have various health consequences for respondents based on their frequency and severity. Therefore, in the current study, by using the above eight questions, we created four outcome variables – any IPV, moderate physical IPV, severe physical IPV, and sexual IPV. The dichotomous outcome variable any IPV, reflects whether a woman reported any positive answer to the eight questions, indicating any kind of the eight forms of violence. Based on the severity of IPV, and following a study by Naved, Azim, Bhuiya, and Persson (23), the experiences of moderate physical IPV and severe physical IPV were measured using questions (a–c) and questions (d–g), respectively. When a woman reported any positive answer to the three questions (a–c), she was considered to have experienced moderate physical IPV. When a woman reported at least one positive response to the four questions (d–g), she was considered to have experienced severe physical IPV. The fourth dichotomous outcome variable, sexual IPV was created to observe having experienced sexual IPV using question number (h). In other words, if a woman gave a positive answer to the question (h), she was considered to have experienced sexual IPV.

### Witnessing IPPV

In BDHS-2007, witnessing IPPV, that is, witnessing domestic violence against mothers perpetrated by fathers, was assessed by asking each respondent whether her father ever hit/beat her mother. The response categories were: 1) yes 2) no 3) don't know. We dropped the cases whose responses were ‘don't know’. Respondents who answered ‘yes’ were considered to have witnessed IPPV, and respondents who marked ‘no’ were considered not to have witnessed IPPV.

### Covariates

Several covariates that have been theoretically and empirically linked to IPV (4, 24, 25) were included in this study. Current age (expressed as group variable: ‘15–24’, ‘25–34’, and ‘35–49’), age at first marriage (expressed as group variable: ‘≤15’, ‘16–17’, and ‘18+’), marital duration (<5 years versus ≥5 years), number of living children (expressed as group variable; ‘≤2’, ‘3–4’, and ‘5+’), place of residence (urban versus rural), literacy status (illiterate versus literate), and work status (yes versus no) were included. Husband's literacy status (illiterate versus literate) is the only variable that contains information about respondents’ husbands. Primary education refers to completing grade 5, secondary education refers to completing grade 10, and higher education refers to education beyond grade 10 in BDHS-2007 (17). Respondents/husbands with no schooling were considered to be illiterate, and with primary, secondary, or higher education as literate.

The wealth index was constructed from data on household assets, including ownership of durable goods (such as televisions and bicycles) and dwelling characteristics (such as sources of drinking water, sanitation facilities, and construction materials), based on the assumption that the wealth of a household is related to an underlying continuum of economic status. To create the wealth index, each asset was assigned a weight (factor score) generated by employing principal component analysis, and the resulting asset scores were then standardized in relation to a normal distribution with a mean of zero and standard deviation of one. Each household was then assigned a score for each asset, and the scores were summed for each household. Individuals were then ranked according to the total score of the household in which they resided, and the sample was divided into quintiles from ‘poorest’ to ‘richest’.

Respondents were asked the following questions to observe their opinion concerning violence against wives: ‘It would be justified if husbands beat them under four circumstances: 1) if she goes outside without telling him, 2) if she neglects the children, 3) if she argues with him, and 4) if she refuses to have sex with him’. If a respondent justified violence according to any of these circumstances, she was assigned a score of 1, and if she justified violence under none of these circumstances, she was assigned a score of 0.

Women's autonomy was measured based on responses to questions regarding who makes decisions according to five circumstances: spending money; health care; making large household purchases; making household purchases for daily needs; visiting family or relatives. For the first circumstance, the response categories were: (a) respondent alone, (b) respondent and husband/partner, (c) respondent and other person, (d) husband/partner alone, (e) someone else. In the rest of the circumstances, however, the option ‘(f) other’ was added. The internal consistency among the five variables (above five circumstances) was acceptable (Cronbach's *α*=0.78) to form an index, according to standards outlined in the literature (26). Respondents’ involvement (options (a), (b), and (c)) in any of the above circumstances resulted in a score of 1, while no involvement (options (d), (e), and (f)) elicited a score 0. The values were then summed up, resulting in a score ranging from 0 to 5. The women's autonomy index was then constructed following the Human Development Index (HDI) made by the United Nations Development Programme (27), using the formula below:

$$\text{Dimension index}=\frac{\text{Actual score}-\text{Minimum score}}{\text{Maximum score}-\text{Minimum score}}$$

The women's autonomy index was a scale variable with scores ranging from 0 to 1 in accordance with the method of the HDI. Following the categorization of HDI, the women's autonomy index was then categorized into three groups as: 1) low level with an index value less than 0.5, 2) moderate level with an index value between 0.5 and 0.79, and 3) high level with an index value equal or higher than 0.8.

### Analytic plan

After describing the study sample, sociodemographic differences in experiencing different types of IPV were assessed by Chi-square tests. Finally, binary logistic regression models were fit to determine the association between witnessing IPPV and different types of IPV perpetrated against women.

Models 1, 3, 5, and 7 established an association between witnessing IPPV and any IPV, moderate physical IPV, severe physical IPV, and sexual IPV, respectively. Results were presented as odds ratios (ORs) with confidence intervals (CIs) and demonstrated the likelihood of experiencing different types of IPV. Models 2, 4, 6, and 8 examined possible mechanisms driving associations by adjusting for sociodemographic characteristics for different types of IPV. Additionally, an interaction between witnessing IPPV and respondent's education, and husband's education for each of these adjusted models were tested and found not to be significant. The net significance of witnessing IPPV in all models was estimated by calculating the difference in the log-likelihood statistic between models that did and did not contain witnessing IPPV (the latter not shown). The analyses were conducted using STATA/SE 12.1 (StataCorp LP, College Station, TX, USA), and a sample weight was applied to accommodate the survey design. In particular, ‘svy’ commands were used to allow for adjustment of the cluster sampling design, sampling weights, stratification, and the calculation of standard errors. These commands used the Taylor series linearization method to estimate CIs around prevalence estimates.

### Ethical considerations

Data collection procedures for the BDHS-2007 were approved by the ORC Macro Institutional Review Board. All participants were asked to provide informed consent after being read a document emphasizing their voluntary participation in the projects.

The questions on IPV in the Women's Questionnaires were administered to only one eligible respondent per household. The interviewers read an additional statement informing the respondents that the questions to follow could be of a sensitive nature and reassuring them of the confidentiality of their responses. Selecting only one person to receive the domestic violence questions protected the privacy of that person and helped ensure that other respondents in the household were not aware of the types of questions that the selected respondent was asked. If privacy could not be ensured, the interviewers were instructed to skip the module.

## Results

Table 1 provides the distribution of the background characteristics of Bangladeshi married women, and the percentages of different types of IPV that were experienced during the 12 months prior to the survey. The average age of the respondents was 30 years, and the mean age at first marriage was 15 years. Although the legal age of marriage for women in Bangladesh is 18 years, 83% of respondents reported being married before reaching age 18. Around one in three respondents were illiterate. Only one-third of respondents were currently working. The majority of respondents (78%) came from rural areas. More than half of the respondents (51%) had either low or moderate autonomy. Twenty-six percent of respondents witnessed their fathers hitting or beating their mothers.

**Table 1:** Characteristics of the study population (*N*=3,910)

Variables	Mean	SD
Age	29.9	8.7
Age at first marriage	15.2	2.6
	Percentage	Numbers
Age group		
15–24	31.8	1,219
25–34	37.1	1,430
35–49	31.1	1,261
Age at first marriage		
≤ 15	62.9	2,300
16–17	20.5	849
18 +	16.6	761
Respondent's literacy status		
Illiterate	33.7	1,216
Literate	66.3	2,694
Marital duration		
Under 5 years	16.4	655
5 years and above	83.6	3,255
Number of living children		
≤ 2	56.3	2,215
3–4	31.8	1,205
5 +	11.9	490
Work status		
No	67.6	2,758
Yes	32.4	1,152
Place of residence		
Urban	22.4	1,459
Rural	77.6	2,451
Husband's literacy status		
Illiterate	36.1	1,278
Literate	63.9	2,632
Women's autonomy		
Low	33.9	1,369
Moderate	17.0	652
High	49.1	1,889
Witnessed IPPV		
No	73.6	2,906
Yes	26.4	1,004
Different types of IPV against women		
Any IPV	24.8	942
Moderate physical IPV	19.2	743
Severe physical IPV	8.9	351
Sexual IPV	11.0	411
Husband slapped	17.8	691
Husband pushed, shook or threw something	11.3	445
Husband physically forced to have sex when not wanted	11.0	411
Husband punched with fist or with something harmful	7.2	277
Husband twisted her arm or pull her hair	6.2	251
Husband kicked, dragged or beat her up	6.1	241
Husband tried to choke or burn	2.7	107
Husband threatened or attacked with knife, gun or any other weapon	0.6	23

SD, standard deviation; IPPV, inter-parental physical violence.

The most common form of IPV was slapping (17.8%) followed by pushing, shaking or throwing something (11.3%), and forcing unwanted sex (11.0%). The least common form of IPV was threatening or attacking with a knife/gun/any other weapons (0.6%). In summary, 25% of respondents experienced some form of IPV, 19% experienced moderate physical IPV, 9% experienced severe physical IPV, and 11% experienced sexual IPV during the 12 months prior to the survey.

Table 2 displays differences in experiencing different types of IPV against married women by selected sociodemographic characteristics in Bangladesh. Results revealed that women who witnessed IPPV had almost twice the likelihood of experiencing all types of IPV (any, moderate physical, severe physical, and sexual IPV) than women who never witnessed IPPV. Younger women (aged 15–24) were significantly more likely to report experiencing all types of IPV than older women. All types of IPV decreased with the increase in age at first marriage for women. All types of IPV also decreased with the increase in marital duration, but it was not statistically significant in the case of severe physical IPV. A higher number of living children were found to be negatively associated with all types of IPV; comparatively women with five or more living children reported experiencing less IPV than women with less than five children. A slightly higher percentage of rural women experienced IPV when compared to urban women.

**Table 2:** Differences in experiencing types of IPV by selected sociodemographic characteristics (*N*=3,910)

	Any IPV, % (95% CI)	Moderate physical IPV, % (95% CI)	Severe physical IPV, % (95% CI)	Sexual IPV, % (95% CI)
Witnessed IPPV				
No	19.8 (17.9–21.7)	14.7 (13.1–16.4)	6.7 (5.7–7.8)	9.0 (7.7–10.5)
Yes	38.9 (35.2–42.7)	31.8 (28.5–35.3)	15.2 (12.7–18.1)	16.5 (14.0–19.4)
*p*	0.00	0.00	0.00	0.00
Age group				
15–24	33.7 (30.1–37.5)	27.3 (24.0–30.7)	12.4 (10.5–14.7)	15.1 (12.8–17.7)
25–34	25.7 (23.2–28.4)	19.5 (17.2–22.0)	8.6 (7.0–10.4)	11.0 (9.3–12.8)
35–49	14.6 (12.4–17.2)	10.7 (8.8–13.0)	5.8 (4.4–7.6)	6.8 (5.4–8.7)
*p*	0.00	0.00	0.00	0.00
AFM				
≤15	27.0 (24.7–29.4)	21.3 (19.3–23.5)	10.3 (8.9–11.8)	12.2 (10.6–14.0)
16–17	25.9 (22.2–30.0)	19.2 (16.2–22.5)	8.7 (6.6–11.3)	10.8 (8.5–13.8)
18+	15.2 (12.3–18.5)	11.3 (8.9–14.3)	4.2 (2.8–6.1)	6.6 (4.6–9.4)
*p*	0.00	0.00	0.00	0.00
Marital duration				
<5 years	31.4 (27.2–36.0)	23.3 (19.6–27.4)	10.3 (8.0–13.2)	15.2 (12.4–18.6)
≥5 years	23.5 (21.6–25.5)	18.4 (16.8–20.2)	8.7 (7.5–10.0)	10.1 (8.8–11.6)
*p*	0.00	0.00	0.17	0.02
NLC				
≤2	27.5 (25.0–30.2)	21.8 (19.6–24.2)	9.8 (8.3–11.5)	11.8 (10.3–13.6)
3–4	22.6 (19.8–25.7)	16.8 (14.6–19.4)	8.0 (6.3–10.1)	10.4 (8.6–12.5)
5+	17.7 (14.2–21.9)	13.5 (10.5–17.2)	7.4 (5.2–10.4)	8.6 (6.1–12.0)
*p*	0.00	0.00	0.04	0.04
Place of residence				
Urban	21.2 (18.4–24.3)	17.4 (14.9–20.2)	8.7 (6.7–11.1)	8.6 (6.9–10.7)
Rural	25.8 (23.5–28.3)	19.7 (17.8–21.9)	9.0 (7.8–10.4)	11.7 (10.1–13.4)
*p*	0.01	0.17	0.57	0.02
LS				
Illiterate	25.4 (22.4–28.7)	19.7 (17.2–22.4)	10.8 (8.9–13.2)	11.4 (9.3–13.8)
Literate	24.5 (22.2–26.9)	19.0 (17.0–21.1)	8.0 (6.8–9.3)	10.8 (9.3–12.4)
*p*	0.02	0.02	0.00	0.25
Work status				
No	23.6 (21.5–25.9)	19.0 (17.1–21.1)	8.8 (7.6–10.3)	10.1 (8.6–11.7)
Yes	27.2 (23.8–31.0)	19.6 (16.7–23.0)	9.1 (7.4–11.2)	12.9 (10.9–15.3)
*p*	0.00	0.37	0.16	0.00
Husband's LS				
Illiterate	28.6 (25.7–31.6)	22.3 (19.8–25.1)	11.2 (9.3–13.3)	13.6 (11.6–16.0)
Literate	22.7 (20.6–24.9)	17.5 (15.6–19.5)	7.7 (6.5–9.1)	9.5 (8.1–11.1)
*p*	0.00	0.00	0.00	0.00
Wealth index				
Poorest	32.3 (29.2–37.8)	25.3 (21.6–29.4)	12.0 (9.5–15.1)	16.1 (13.1–19.7)
Poorer	28.3 (24.5–32.5)	21.5 (18.0–25.5)	11.2 (8.6–14.4)	12.6 (9.9–15.9)
Middle	25.1 (21.5–29.1)	20.8 (17.7–24.4)	9.3 (7.1–11.9)	9.9 (7.7–12.7)
Richer	23.0 (19.4–27.0)	19.1 (15.7–23.0)	8.4 (6.4–10.9)	9.2 (7.0–12.0)
Richest	13.2 (10.5–16.5)	8.6 (6.5–11.2)	3.4 (2.1–5.5)	6.6 (4.8–9.0)
*p*	0.00	0.00	0.00	0.00
Wife beating justified				
No	22.9 (20.8–25.2)	17.1 (15.3–19.1)	8.0 (6.8–9.4)	10.5 (9.0–12.1)
Yes	29.0 (26.0–32.1)	24.0 (21.4–26.9)	11.1 (9.4–13.1)	12.2 (10.0–14.7)
*p*	0.00	0.00	0.00	0.01
Women's autonomy				
Low	27.1 (24.0–30.5)	20.1 (17.6–22.7)	9.0 (7.5–10.7)	13.0 (10.8–15.6)
Moderate	24.8 (21.1–28.9)	20.9 (17.4–24.8)	10.7 (8.0–14.6)	11.5 (9.1–14.5)
High	23.2 (20.8–25.7)	18.0 (16.0–20.3)	8.2 (6.9–9.9)	9.4 (7.9–11.1)
*p*	0.08	0.10	0.21	0.01

IPV, intimate partner violence; CI, confidence interval; AFM, age at first marriage; NLC, number of living children; LS, literacy status; IPPV, inter-parental physical violence. p Values are of the Chi-square tests.

Illiterate women experienced all types of IPV at slightly higher rates than literate women. Working women reported experiencing all kinds of IPV at slightly higher rates than women who were not currently working, but it was not statistically significant in respect to moderate and severe physical IPV. Women with illiterate husbands experienced more violence than women with literate husbands. Interestingly, all forms of IPV decreased significantly with increasing wealth.

Women who thought wife beating is justified experienced all types of IPV at higher percentages than their counterparts. Women with high autonomy experienced less IPV than their counterparts.

Table 3 provides the results of logistic regression that predicted the odds of having different types of IPV, focusing initially on the association between witnessing IPPV and IPV. The table reveals that, without adjusting for additional controls, women who witnessed IPPV were more likely to experience all types of IPV: OR=2.6 (95% CI: 2.2–3.1) for Model 1, OR=2.7 (95% CI: 2.2–3.3) for Model 3, OR=2.5 (95% CI: 2.0–3.2) for Model 5, and OR=2.0 (95% CI: 1.6–2.5) for Model 7. The association between witnessing IPPV and IPV is attenuated, but persists after controlling for the covariates in Models 2, 4, 6, and 8. Women who witnessed IPPV were 2.4 (95% CI: 2.0–2.8) times more likely to experience any IPV (Model 2), 2.5 (95% CI: 2.0–3.0) times more likely to experience moderate physical IPV (Model 4), 2.3 (95% CI: 1.8–3.0) times more likely to experience severe physical IPV (Model 6), and 1.8 (95% CI: 1.4–2.3) times more likely to experience sexual IPV (Model 8).

**Table 3:** Association between witnessing IPPV and different types of IPV against women

	Any IPV		Moderate physical IPV		Severe physical IPV		Sexual IPV	
				
	Model 1	Model 2	Model 3	Model 4	Model 5	Model 6	Model 7	Model 8
	OR (95% CI)	OR (95% CI)	OR (95% CI)	OR (95% CI)	OR (95% CI)	OR (95% CI)	OR (95% CI)	OR (95% CI)
Witnessed IPPV (No^R^)								
Yes	2.6* (2.2–3.1)	2.4* (2.0–2.8)	2.7* (2.2–3.3)	2.5* (2.0–3.0)	2.5* (2.0–3.2)	2.3* (1.8–3.0)	2.0* (1.6–2.5)	1.8* (1.4–2.3)
Age group (15–24^R^)								
25–34		0.7† (0.5–0.9)		0.6† (0.4–0.8)		0.6† (0.4–0.9)		0.7 (0.5–1.0)
35–49		0.3* (0.2–0.5)		0.3* (0.2–0.4)		0.4* (0.2–0.6)		0.4* (0.3–0.7)
AFM (<16^R^)								
16–17		1.1 (0.8–1.3)		1.0 (0.8–1.2)		1.0 (0.7–1.3)		0.9 (0.7–1.3)
18 +		0.6† (0.5–0.8)		0.6† (0.5–0.8)		0.5† (0.3–0.8)		0.6§ (0.4–1.0)
LS (literate^R^)								
Illiterate		1.1 (0.9–1.4)		1.1 (0.9–1.4)		1.5§ (1.1–2.0)		1.0 (0.7–1.4)
Work status (No^R^)								
Yes		1.1 (0.9–1.4)		0.9 (0.7–1.1)		0.9 (0.6–1.2)		1.3§ (1.0–1.7)
Husband's LS (literate^R^)								
Illiterate		1.1 (0.9–1.4)		1.1 (0.9–1.4)		1.1 (0.8–1.5)		1.3 (1.0–1.7)
Wealth index (poorest^R^)								
Poorer		0.8 (0.7–1.1)		0.9 (0.6–1.1)		1.0 (0.7–1.4)		0.8 (0.6–1.2)
Middle		0.7§ (0.6–0.9)		0.8 (0.6–1.1)		0.8 (0.5–1.2)		0.7§ (0.5–1.0)
Richer		0.7§ (0.5–0.9)		0.8 (0.6–1.1)		0.8 (0.5–1.2)		0.7 (0.4–1.0)
Richest		0.4* (0.3–0.5)		0.3* (0.2–0.4)		0.3* (0.2–0.5)		0.5† (0.3–0.9)
Wife beating justified (No^R^)								
Yes		1.2§ (1.0–1.5)		1.3§ (1.1–1.6)		1.2 (1.0–1.6)		1.1 (0.8–1.4)
Women's autonomy (high^R^)								
Low		1.1 (0.9–1.4)		1.0 (0.8–1.2)		1.0 (0.8–1.3)		1.4† (1.0–1.8)
Moderate		1.1 (0.8–1.4)		1.2 (0.9–1.5)		1.3 (0.9–2.0)		1.3 (0.9–1.7)
L	−2081.5	−1959.6	−1834.2	−1716.3	−1148.8	−1083.6	−1284.0	−1235.8
Δ-2X LL^a^	154.8*	119.8*	134.2*	103.7*	64.2*	48.5*	60.9*	44.1*

Model 2, 4, 6, and 8 were adjusted for the effects of marital duration, number of living children, and place of residence.

IPV, intimate partner violence; IPPV, inter-parental physical violence; OR, odds ratios; CI, confidence interval; AFM, age at first marriage; LS, literacy status; L, log likelihood

R Reference category.

* *p*<0.001;

† *p*<0.01;

§ *p*<0.05.

a Change in Δ-2X log-likelihood when adding IPPV to a model containing other variables. For Model 1, 3, 5, and 7, it was the change when adding IPPV to a model containing the constant only.

Similar findings were observed for age groups in both the Chi-square tests and logistic regression analyses; women aged 15–24 were more likely to experience all types of IPV than women aged 25 and above. Women who married at age 18 or above were less likely to experience all types of IPV than women whose age at marriage was less than 18 years. Illiterate women were 1.5 times more likely to experience severe physical IPV than literate women. Working women were 1.3 times more likely to experience sexual IPV. Women from the wealthiest families were less likely to experience all types of IPV when compared to women from the poorest families.

Women who think wife beating is justified were more likely to experience any IPV and moderate physical IPV. Women with low autonomy were 1.4 times more likely to experience sexual IPV than women with high autonomy.

The Δ-2X LL values were statistically significant for all the models and indicated that the equations showing the relationship between witnessing IPPV and IPV with additional controls were better fit than the equations that did not capture witnessing IPPV.

## Discussion

Our study indicates that witnessing IPPV was positively associated with all types of IPV against women in Bangladesh. One-fourth of women witnessed IPPV and experienced any IPV and women who ever witnessed IPPV were significantly more likely to experience any moderate physical, severe physical, and sexual IPV than women who never witnessed IPPV. During regression analysis we also tested for the most important correlates of IPV and found that witnessing IPPV was the only covariate to demonstrate a strong, consistent relationship with IPV across models.

Parents are the primary socialization agents involved in children's emotional and overall development. Children learn by watching their parents and imitating their behavior from infancy through young adulthood. As Bangladesh is a highly patriarchal society and mired by gender inequalities in both public and private spheres, male children are supported throughout their lives. Female children face lowered expectations for educational attainment and economic success and are taught to have tolerant attitudes toward these imbalances. In theory, internalization of patriarchy is more likely under family systems that 1) ascribe women to be financially dependent and obedient, 2) ascribe men to be providers and enforcers of obedience, and 3) promise benefits to compliant women (28). In Bangladesh, boys are directed to participate in the activities other than childcare and cooking, particularly those involving risk, since it is believed that overcoming obstacles will better prepare them for roles as fathers, husbands, and family caretakers. On the contrary, female children are instructed in childcare and household management, a reflection on lowered expectations concerning female roles in the public sphere. Therefore, boys and girls in Bangladesh learn to be tolerant of violence directed at women by watching IPPV and come to see gender bias as normal. Our findings revealed that witnessing IPPV increases the probability of experiencing IPV, which suggests that IPV passes from one generation to another (i.e. from a woman's family of origin to her own family) in Bangladesh. Therefore this study stresses interventions that can prevent IPPV so that the next generation can enjoy a happy married life without exposure to violence. This may become possible through educational programs that increase awareness about the nature and consequences of IPV and motivate married-couples, especially men since they are more likely to be perpetrators, to recognize and prevent abusive behavior from entering their households.

Our results are consistent with the literature (4, 29) that older women are less likely to experience IPV than younger women. A general assumption is that as women grow older, their roles as mothers become more prominent than their roles as wives, and they achieve a certain status at the household and community levels which reduces the likelihood of violence (7). Women whose age at first marriage was less than 18 were more likely to experience all types of IPV than women whose age at first marriage was 18 and above. Legal age at marriage for girls is 18 years in Bangladesh. However, many marriages are arranged for girls when they are teenagers (30). In developing countries around the world, it has been found that girls who marry as adolescents have lower educational attainment, lower social status in their husbands’ families, and experience higher rates of domestic violence (31). Therefore, strictly enforcing the existing laws of age at marriage for women (18 years and above) and/or increasing age at marriage might be a good step toward reducing all types of IPV against women in Bangladesh.

Our findings are also consistent with the literature (5, 6) in that illiterate women and women with illiterate husbands experienced higher rates of IPV than their counterparts. Education might confer social empowerment and greater women's autonomy, which in turn could help change social norms, improve socioeconomic conditions (32, 33), and help reduce IPV. This study therefore recommends providing education to illiterate married couples.

Education may increase women's opportunities for wage labor and augment incomes. Consequently, the economic conditions of households with working women tend to have better resources than compared to households without working women. However, our study revealed that working women were more likely to experience sexual IPV than non-working women. Since poor households are comprised of a higher percentage of working women than wealthy households (results not shown), working women might experience higher IPV compared to non-working women. The findings are in line with past research, which has found (32, 34) that changes in women's status through education or increased earnings can place women at risk for violence in communities where the status of women is traditionally very low. Formal education as well as training in vocational skills should help a couple become wage earners. Whereas the economic conditions of the household would improve, the couple would have less time to spend together. Status as wage earners may enhance women's position in respect to household management, but may also result in unforeseen conflict, as may be the case with the poorest households in this study's sample.

Previous research has also found that women from the poorest households are more likely to experience IPV than women from wealthier households (3, 4, 30). Furthermore, justification of beating on the part of wives was associated with an increased risk of IPV in the sample, which is in agreement with a study by Kishor and Johnson (7). Similarly, women with low autonomy had increased risk of IPV when compared to women with moderate/high autonomy. Therefore, empowering women through pragmatic education and increasing women's participation in every household decision is the recommendation of this study. Furthermore, educational programs should stress the vital role assumed by male household members in counteracting abusive relationships as well as encouraging females’ participation in decision making, autonomy, and education.

Some limitations should be considered when interpreting our findings. First, these data were cross-sectional. Therefore, causal relationships cannot be inferred from the analyses. We were careful to select predictors of IPV based on the previous literature and that complemented our hypotheses as well as the resulting analytic plan. However we readily admit than no model, and in particular cross-sectional models, can be complete in explaining the outcome variable. After running successive (stepwise) preliminary models, we adjusted for factors based on the study's theoretical foundation, concerns over multicollinearity and the power of the covariates in explaining IPV. Second, this study is restricted to currently married women. For this reason the prevalence of witnessing IPPV and IPV should not be generalized to the female population of Bangladesh. Third, ‘witnessing IPPV’ in the DHS surveys is derived from the question ‘As far as you know, did your father ever beat your mother?’ This should be literally interpreted as whether the respondents knew that their father abused their mother. But, an intuitive question ‘How did a respondent know about her father's violence against her mother?’ could also be answered as ‘having witnessed’ or ‘someone informs the respondent’. It is unlikely that without witnessing IPPV or recalling the results of violence that the respondent would credit evidence from an informant that her father ever beat her mother. And, we believe it unlikely that a respondent would say ‘yes’ to an interviewer in response to the question ‘As far as you know, did your father ever beat your mother?’ without witnessing IPPV. Thus, it seems reasonable that the respondents to this study witnessed and knew about this violence. Moreover, utilizing the Nigerian Demographic and Health Survey-2008 data, a study by Uthman, Moradi, and Lawoko (16) used the term ‘Witnessed physical violence in childhood’ for the same question, ‘As far as you know, did your father ever beat your mother?’ Fourth, variable selection was constrained by preexisting data; some key indicators were unavailable in BDHS-2007, such as type of marriage – arranged marriage/love marriage, demand/dowry at the time of marriage. Demand/dowry is an important issue related to violence against women in the Bangladeshi context. Fifth, the study looks at the intergenerational transmission of IPV only from the woman's family perspective because the information on husbands’ attitudes toward violent acts and whether husbands had witnessed IPPV is missing in the data set; similar analyses for the perpetrator dimension, that is, the husbands’ family perspective and a comparative study between male and female respondents would clearly help understand the perpetuation of IPV in the patriarchal society of Bangladesh. Finally, the possibility of under-reporting must be considered. Many women may not disclose incidence of domestic violence because they fear further IPV or because of shame or embarrassment (17).

The study has some strengths as well. The field staff of BDHS-2007 was provided with training to implement the domestic violence module so that they could collect violence data in a secure, confidential, and ethical manner that facilitated a safe atmosphere for respondents to comfortably discuss this issue (17). The data came from a nationally representative survey, and a relevant subset was extracted consisting of currently married women aged 15–49.

Despite these limitations, our results have potentially important implications for policy makers and planners to reduce all types of IPV in Bangladesh. Special focus should be given to reducing and eliminating IPPV so that future generations can enjoy a happy married life without exposure to IPV in Bangladesh. Further exploratory and action research are needed to address the ways to reduce IPV most effectively and efficiently in Bangladesh. Finally, longitudinal research on the relationship between witnessing IPPV and IPV is needed to better understand generational transference of IPV.
